# Thymoquinone loaded on chitosan nanoparticles alleviated the consequences of cryptosporidiosis infection in a murine model: Evidence from parasitological, histopathological, immunohistochemical, and immunological studies

**DOI:** 10.1371/journal.pone.0325077

**Published:** 2025-05-30

**Authors:** Abdelmoneim A. Ali, Eman S. El-Wakil, Al-sayed R. Al-Attar, Mahmoud Fawzy, Aya Samy, Zienab E. Eldin, Wafa Abdullah I. Al-Megrin, John T. Nazeer

**Affiliations:** 1 Pathology Department, Faculty of Veterinary Medicine, Zagazig University, Zagazig, Egypt; 2 Department of Parasitology, Theodor Bilharz Research Institute, Giza, Egypt; 3 Materials Science and Nanotechnology Department, Faculty of Postgraduate Studies for Advanced Sciences (PSAS), Beni-Suef University, Beni-Suef, Egypt; 4 Department of Biology, College of Science, Princess Nourah bint Abdulrahman University, Riyadh, Saudi Arabia; 5 Department of Medical Parasitology, Faculty of Medicine, Ain Shams University, Cairo, Egypt; Mansoura University Faculty of Veterinary Medicine, EGYPT

## Abstract

**Background:**

Cryptosporidiosis, a parasitic zoonosis caused by the genus *Cryptosporidium* (*C.*), currently lacks a vaccine or fully effective treatment. Nitazoxanide (NTZ), the only medication approved by the US Food and Drug Administration for treating cryptosporidiosis, exhibits limited efficacy in immunosuppressed hosts. Thymoquinone (THQ), the active component of *Nigella sativa*, possesses immunomodulatory, antitumor, hepatoprotective, antioxidant, antimicrobial, and antiprotozoal properties. This study evaluated the therapeutic effects of THQ alone or loaded onto chitosan nanoparticles (CsNPs) against *Cryptosporidium parvum* infection compared to NTZ.

**Methods:**

Chitosan nanoparticles were synthesized and characterized using X-ray diffraction (XRD), Fourier transform infrared spectroscopy (FTIR), zeta potential analysis, and scanning electron microscopy. The cytotoxicity of CsNPs, THQ/CsNPs, and NTZ/CsNPs was evaluated on HT-29 cells. Mice were divided into seven groups to assess treatment efficacy through parasitological analysis of oocyst shedding, histopathological examination of intestinal, hepatic, and splenic tissues, immunohistochemical analysis using cyclin D1 staining of intestinal tissue, and immunological analysis measuring IFN-γ and IL-10 cytokine levels. Additionally, pharmacokinetic profiles of THQ and NTZ in free and nanoparticle-loaded forms were analyzed.

**Results:**

XRD confirmed changes in peak position, shape, and intensity following the loading of THQ and NTZ into CsNPs. FTIR spectra demonstrated distinct differences in peak patterns between loaded nanoparticles and individual components, confirming successful drug encapsulation. Moreover, cytotoxicity studies showed dose-dependent effects on cell viability, with NTZ/CsNPs exhibiting the highest cytotoxicity. Regarding oocyst shedding reduction, THQ demonstrated greater efficacy than NTZ (77% vs. 54%), which was further enhanced when loaded onto CsNPs (89% for THQ/CsNPs vs. 78% for NTZ/CsNPs). Histopathological analysis revealed the restoration of structural alterations in intestinal, hepatic, and splenic tissues in treated groups. Cyclin D1 immunohistochemical staining showed a significant reduction in immunoreactivity in the THQ/CsNP-treated group compared to other treatments. Furthermore, immune responses were modulated by nanoparticle therapies, with significantly lower IFN-γ levels and higher IL-10 levels in treated groups. Pharmacokinetic analysis demonstrated that CsNP formulations significantly improved drug bioavailability by achieving higher peak plasma concentrations (*C*_max_), earlier time to peak concentration (*T*_max_), and prolonged half-life (*t*_1/2_) compared to free drugs.

**Conclusion:**

Thymoquinone demonstrated significant potential as an anti-cryptosporidiosis therapeutic agent, with enhanced efficacy when loaded onto chitosan nanoparticles. Chitosan-based nanoparticle formulations improved the pharmacokinetic profiles of both THQ and NTZ, offering a promising strategy for enhancing drug bioavailability and retention while reducing parasitic burden and modulating immune responses effectively.

## Introduction

Cryptosporidiosis, a prevalent zoonotic parasitic disease caused by the genus *Cryptosporidium* (*C.*), has a global distribution. Although over forty *Cryptosporidium* species have been identified, most cryptosporidiosis cases are caused by *C. hominis* and *C. parvum* [1]. Infection occurs through ingesting oocysts in contaminated food or water excreted by infected individuals or animals. Although the parasite’s development is primarily confined to the intestine, immunosuppressed hosts may exhibit involvement of the biliary system, pancreas, and lungs [2].

Cryptosporidiosis induces gastrointestinal complications, including severe diarrhea. It causes acute, self-limiting diarrhea in immunocompetent hosts and fatal diarrhea in immunocompromised hosts, infants, and young animals [3].

One of the most significant issues affecting economic productivity and animal welfare in the beef, goat, sheep, and dairy industries is infectious diarrhea in young ruminants, with *C. parvum* being one of the primary pathogens responsible [4].

*Cryptosporidium* species in the epithelial lining of the gastrointestinal tract induce various pathological alterations. These include submucosal edema, loss of the intestinal microvillus border, villi shortening, crypt lengthening, and mononuclear cell inflammatory infiltration in the lamina propria, resulting in acute gastrointestinal disruption [5].

Moreover, there is a general correlation between cancer and chronic inflammation, with *C. parvum* being associated with an increased risk of developing colorectal cancer [6].

Despite evaluating over 200 substances for their anti-cryptosporidial activity in humans and animals, no vaccine or effective medication is available [7].

The sole drug approved by the Food and Drug Administration (FDA) in the United States for treating *C. parvum*-induced infectious diarrhea, particularly in immunocompetent patients, is nitazoxanide (NTZ) [8]. NTZ is a thiazolide compound with broad-spectrum anti-helminthic and antiprotozoal properties. However, the host’s immune status can influence NTZ’s therapeutic efficacy [9]. NTZ showed poor outcomes in immunocompromised hosts, while in immunocompetent patients, it produced only transient effects with frequent relapses [10]. These limitations have driven research into the development of innovative and alternative therapies. Furthermore, novel and sustained interventions remain necessary to combat this disease, particularly in vulnerable individuals [11].

Thymoquinone (THQ), a volatile oil derived from *Nigella sativa* (*N. sativa*) seeds, accounts for most of the biological properties of *N. sativa*. Several researchers have documented the diverse pharmacological characteristics of THQ, including its immunomodulatory, antitumor, hepatoprotective, antihistaminic, gastroprotective, antioxidant, nephroprotective, and antimicrobial activities [12]. However, its clinical application is limited by its lipophilic nature, poor aqueous solubility, and rapid degradation, which result in low bioavailability and reduced therapeutic efficacy. Nanotechnology provides an innovative approach to address these challenges by encapsulating THQ within nanoparticles to enhance its stability, solubility, and controlled release [13].

Chitosan (Cs) is a naturally occurring polymer produced by deacetylating chitin in an alkaline medium. It is a linear polysaccharide primarily found in the cuticular exoskeletons of shrimp and other crustaceans and is composed of 1,4-linked glucosamine and N-acetylglucosamine [13]. Cs is one of the most widely used polysaccharides for encapsulating active compounds such as terpenes, essential oils, and various medications due to its structure-forming ability, biocompatibility, biodegradability, high stability, and low toxicity, making it suitable for pharmaceutical applications [14]. Its mucoadhesive properties enable adhesion to mucosal surfaces, prolonging drug residence time and enhancing absorption [15].

Among various nanoparticle formulations, chitosan nanoparticles (CsNPs) have emerged as a preferred drug carrier due to their biocompatibility, biodegradability, mucoadhesive properties, and ability to improve intestinal permeability. These features make CsNPs particularly effective for delivering therapeutic agents such as THQ to intracellular parasites like *Cryptosporidium* [12,16]. Additionally, CsNPs can transiently open tight junctions in epithelial cells, improving drug permeability across biological barriers [17]. These nanoparticles provide controlled and sustained drug release, enhancing therapeutic efficacy while minimizing side effects. Furthermore, CsNPs are easy to prepare and can encapsulate many drugs, including hydrophobic compounds like thymoquinone, improving their stability and bioavailability [18].

It is crucial to identify specific markers, as dysplastic alterations in the gastrointestinal tract may result from inflammation and infection. A significant number of colorectal tumors, particularly those with high-grade dysplasia, exhibit markedly aberrant expression of retinoblastoma protein, cyclins D1, D2, and the cyclin-dependent kinase inhibitor p16. One such marker is the essential protein cyclin D1, which regulates the cell cycle. Overexpression of this protein disrupts normal cell cycle regulation, contributing to cancer pathophysiology. Since cyclin D1 level assessment is reproducible, it can be considered an effective prognostic indicator [19].Unlike general inflammatory markers (e.g., CD68, IL-6, TNF-α), Cyclin D1 is directly involved in cell proliferation and oncogenesis. Its overexpression is often seen in dysplastic and neoplastic cells, making it more specific for identifying pre-malignant changes that may be a consequence of *C. parvum* infection [20].

While THQ’s antiparasitic effects have been explored against other protozoal pathogens such as *Babesia* [21], and *Leishmania* [22,23], its application against *Cryptosporidium* in a nanoparticle-encapsulated form represents a novel approach. This study is unique in investigating the comparative efficacy of THQ-loaded chitosan nanoparticles versus THQ alone and NTZ in an immunocompromised mouse model. By leveraging nanotechnology to enhance THQ’s stability and bioavailability, this research aims to provide innovative insights into alternative therapeutic strategies for cryptosporidiosis.

Based on this background, the current study hypothesizes that thymoquinone encapsulated in chitosan nanoparticles may demonstrate potential therapeutic efficacy compared to thymoquinone alone and nitazoxanide in treating *Cryptosporidium parvum* infection in an immunocompromised mouse model.

## Materials and methods

### Preparation of drugs

Thymoquinone (THQ), nitazoxanide (NTZ), chitosan (Cs, medium-molecular weight; M.W. 75 kDa), and tripolyphosphate (TPP) were purchased from Sigma-Aldrich Chemical Co. (St. Louis, MO, USA). Glacial acetic acid and dimethyl sulfoxide (DMSO) were obtained from Merck (Darmstadt, Germany). All other solvents and reagents were of analytical reagent grade.

### Synthesis of chitosan nanoparticles (CsNPs), thymoquinone-loaded chitosan nanoparticles (THQ/CsNPs), and nitazoxanide-loaded chitosan nanoparticles (NTZ/CsNPs)

CsNPs were created with minor adjustments using the previously described ionic gelation technique [24]. Briefly, 300 mg of chitosan was dissolved in 100 mL of 1% acetic acid solution. This was stirred at room temperature for 20 min at 1200 rpm to obtain a clear solution. The Cs solution was then sonicated at pH 5 before slowly adding 1 mg/mL of TPP solution under continuous stirring; this induced ionic crosslinking and the formation of CsNPs. For THQ/CsNPs and NTZ/CsNPs, 150 mg of thymoquinone was dissolved in 10 mL of DMSO, and 100 mg of nitazoxanide was dissolved in 8 mL of DMSO. Each drug was added to the preformed CsNPs suspension. These mixtures were stirred for six hours to allow drug encapsulation. The nanoparticles were obtained by cooling centrifugation for 45 min at 14,000 rpm, washed with distilled water to remove any non-encapsulated drug, and lyophilized for 48 hours to obtain dried CsNPs, THQ/CsNPs, and NTZ/CsNPs using a freeze dryer [25].

### Characterizations

#### X-ray diffraction (XRD).

Analysis utilizing X-ray diffraction (XRD) provided insights into the crystalline structure of the analyzed materials. A Cu Kα X-ray source with a wavelength of 1.54 Å was used. The XRD instrument was operated at a 40 kV voltage, and a 30-mA current, resulting in a power output of 1200 W. Data were collected over a 2theta angle range from 10° to 70° at a scanning speed of 2° per minute. The step size was set to 0.050°, with a 1.5-second step time.

#### Fourier transformation infrared spectroscopy (FTIR).

The FTIR technique detected the characteristic functional groups in the materials over the mid-infrared region from 4500 to 500 cm^-1^ wavenumbers. The spectral peaks were analyzed to identify specific molecular structures. FTIR spectra were acquired using a Bruker Vertex 70 and were collected for CsNPs, pure THQ, pure NTZ, THQ/CsNPs, and NTZ/CsNPs. The spectrometer scan resolution was set to 1 cm^-1^. Each sample spectrum represented the average of 3 scans to enhance the signal-to-noise ratio.

#### Determination of hydrodynamic size, polydispersity index (PDI), and zeta (ζ) potential.

Dynamic light scattering (DLS) using a Malvern Zetasizer ZS90 device was utilized to measure key properties of the nanoparticle suspensions, including hydrodynamic size, zeta (ζ) potential, colloidal stability, and polydispersity index. DLS detects variations in scattered light intensity caused by the nanoparticles’ Brownian motion. The analysis was performed on 1 mL samples of CsNPs, pure THQ, pure NTZ, THQ/CsNPs, and NTZ/CsNPs dispersed in distilled water. Triplicate measurements were taken for each sample to ensure the accuracy of the results.

#### Nanoparticle morphology.

The prepared THQ/CsNPs and NTZ/CsNPs were examined using field emission scanning electron microscopy (FESEM, Philips-XL30 equipment, Netherlands) to determine their morphology and particle size.

#### Thymoquinone (THQ) and nitazoxanide (NTZ) entrapment efficiency (EE) and loading capacity (LC).

To determine the efficiency of THQ and NTZ trapping, THQ/CsNPs and NTZ/CsNPs were centrifuged for 40 min at 4°C and 14,000 rpm to separate the supernatant containing non-encapsulated THQ and NTZ. The amount of free THQ and NTZ in the supernatant was then quantified by measuring the absorbance at 257 and 222 nm, respectively, using a UV-visible spectrophotometer. Measurements were performed in triplicate for each formulation batch to ensure statistical reliability [26]. THQ and NTZ entrapment efficiency and loading capacity were calculated using the following equations [27]:


$$\begin{array}{c} \text{THQ}\left(EE\%\right)=THQtotal-THQfree/THQtotal\times 100\% \end{array}$$ (1)

$$\begin{array}{c} \text{THQ}\left(EE\%\right)=THQtotal-THQfree/THQtotal\times 100\% \end{array}$$ (2)

$$\begin{array}{c} THQ\left(LC\%\right)=THQtotal-THQfree/THQCsNPstotalamount\times 100\% \end{array}$$ (3)

$$\begin{array}{c} NTZ\left(LC\%\right)=NTZtotal-NTZfree/NTZCsNPstotalamount\times 100\% \end{array}$$ (4)

For accuracy, the UV-visible spectrophotometer was calibrated with standard solutions of THQ and NTZ to establish a linear relationship between absorbance and concentration. Representative samples from each batch were analyzed, and the mean values and standard deviations were reported to confirm consistency across different formulations.

#### In vitro release study of THQ and NTZ.

The THQ release profile from THQ/CsNPs and NTZ from NTZ/CsNPs were evaluated using a dialysis method [28]. Briefly, 3 mL of the THQ/CsNP suspension was placed in a dialysis bag (M.W. 12 kDa, Sigma–Aldrich). Each sealed dialysis bag was immersed in 50 mL of PBS (pH 6.8) in a shaking incubator at 37 ± 0.5°C and 100 rpm. Samples were withdrawn from the release medium at specified intervals and replaced with fresh buffer to maintain sink conditions. The amount of THQ and NTZ released was measured using a spectrophotometer at 257 and 222 nm, respectively. As a control, the release of free THQ and free NTZ was measured under the same conditions. All measurements were performed in triplicate.

#### Cell culture.

HT-29 cells were obtained from the Tissue Culture Unit of the Holding Company for Biological Products and Vaccines (VACSERA) in Giza, Egypt, and cultured in DMEM supplemented with 10% fetal bovine serum (FBS), 5% glutamine, and 100 U/mL penicillin-streptomycin. The cells were incubated at 37°C in a humidified atmosphere containing 5% CO_2_. Upon reaching 85% confluence, the cells were detached using 0.25% trypsin and subcultured into 25 cm^2^ flasks or 96-well plates based on the experimental needs.

#### Evaluation of cytotoxicity.

HT-29 cells were utilized to evaluate the cytotoxic effects. CsNPs, THQ/CsNPs, and NTZ/CsNPs were suspended in DMEM and serially diluted to 25, 50, 100, 200, 400, and 1000 μg/mL concentrations. The nanoparticle suspensions were sonicated at room temperature at 40 W for 15 minutes to prevent agglomeration before treatment. Untreated HT-29 cells served as the control group.

For the cytotoxicity assessment, the MTT assay was performed as described by [29] to evaluate mitochondrial function and cell viability. HT-29 cells were seeded into 96-well plates at a density of 1 × 10^4^ cells per well and incubated at 37°C for 24 hours to allow attachment before exposure to CsNPs, THQ/CsNPs, and NTZ/CsNPs. The cells were then treated with various concentrations of CsNPs, THQ/CsNPs, and NTZ/CsNPs and incubated for an additional 24 hours at 37°C.

In parallel, HT-29 cells were treated with serial concentrations of the anticancer drug doxorubicin under identical conditions (37°C and 5% CO_2_) for comparison. Untreated control cells received only culture medium without CsNPs, THQ/CsNPs, and NTZ/CsNPs.

After the treatment period, the medium was replaced with fresh medium containing MTT solution, and the plates were incubated at 37°C for 3 hours to allow the formation of purple formazan crystals. The formazan product was solubilized in isopropanol, and the plates were centrifuged at 2300 × g for 5 minutes to separate cell debris and residual nanoparticles. Subsequently, 100 μL of the supernatant was transferred to a new plate, and absorbance was measured at 570 nm using a microplate reader (ELx800, BioTek, USA). All experiments were performed in triplicate.

**Animals:** The study was conducted following approval by the Research Ethics Committee of Zagazig University, Institutional Animal Care and Use Committee (ZU-IACUC), under protocol number ZU-IACUC/2/F/53/2024, following the National Institutes of Health guidelines for animal experimentation. Seventy male laboratory-bred Swiss albino mice free of parasites were included in this study. The mice weighed 20–25 grams at the start of the trial and were between four and six weeks old. They were purchased from the Biological Supply Center of the Theodor Bilharz Research Institute (TBRI). Throughout the trial, the mice were housed in an air-conditioned animal facility maintained at 20–22°C and were provided with a standard commercial pelleted diet and free access to water.

**Experimental design:** The animals, after immunosuppression, were randomly divided into seven groups (each containing 10 mice):

- **Group I**: Immunosuppressed, non-infected, and non-treated (control negative) (GI).
- **Group II**: Immunosuppressed, infected, and untreated (control positive) (GII).
- **Group III**: Immunosuppressed, infected, and treated with chitosan nanoparticles (GIII).
- **Group IV**: Immunosuppressed, infected, and treated with nitazoxanide (GIV).
- **Group V**: Immunosuppressed, infected, and treated with nitazoxanide loaded on chitosan nanoparticles (GV).
- **Group VI**: Immunosuppressed, infected, and treated with thymoquinone (GVI).
- **Group VII**: Immunosuppressed, infected, and treated with thymoquinone loaded on chitosan nanoparticles (GVII).

Furthermore, the study was conducted in a blinded manner, with the animal groups and administered drugs labeled by codes that were only revealed after euthanasia.

**Immunosuppression:** The mice were subjected to immunosuppression by administering dexamethasone (Dexazone, Kahira Pharmaceuticals, Cairo, Egypt) orally through an esophageal tube at a rate of 0.25 µg/g/day for 14 consecutive days before being inoculated with *C. parvum* oocysts. Throughout the experiment, the mice received the same dosage of dexamethasone [30].

**The infection:** In a previous study, we isolated *Cryptosporidium* oocysts from diarrheal calves and genetically identified them as *C. parvum* [31]. The 2.5% potassium dichromate solution containing purified oocysts was stored at 4°C until needed. After preparing an infectious inoculum and counting the number of oocysts in the concentrated stock inoculum, the fluid volume of the inoculum per mouse was determined [32]. Oral-gastric gavage was used to infect each mouse orally with *C. parvum* oocysts. Approximately 3 × 10^3^ oocysts/mouse were used to infect each mouse at 0 days post-infection (dpi) [33].

**Drug regimens:** Nitazoxanide was administered orally at 100 mg/kg/day for five consecutive days to immunosuppressed mice starting at seven dpi [34,35] for Group IV. Nitazoxanide loaded on chitosan nanoparticles was given at the same dose for Group V. Thymoquinone was administered orally at 200 mg/kg three times a week, starting at seven dpi for 2 weeks [36] for Group VI. Thymoquinone loaded on chitosan nanoparticles was given at the same dose for Group VII. Chitosan was administered at 200 mg/kg three times a week, starting at seven dpi for 2 weeks for Group III [36].

**Assessments of the drug therapy**: ***Parasitological examination*:** The mice feces were collected at 7, 14, and 21 dpi. For the oocyst shedding assessment, the number of *Cryptosporidium* oocysts in the fecal pellets was counted after staining with Kinyoun’s acid-fast stain. Fecal samples were collected from each infected mouse. The mean number of oocysts was calculated for each mouse group. After weighing, a milligram of the fecal pellet was placed in 1 milliliter of 10% formalin. Centrifugation concentrated the fecal solution for ten minutes at 7000 rpm. The number of oocysts in one milliliter of the feces sample was calculated using 100 µl of fecal material dried, stained, and observed through an oil immersion lens (x100) [37]. The oocyst count was detected and expressed per gram of feces [38]. The effectiveness of each drug was assessed according to [35] and represented as a percentage of reduction using the following formula:


$$\begin{array}{cc} Efficacy(\%)= & 100\times ((\text{infected model group oocyst count}-\text{treated model group oocyst count})/\\ \; & (\text{infected model group oocyst count})) \end{array}$$

### Histopathological examination

At 21 dpi, the mice were humanely euthanized under light anesthesia induced by isoflurane inhalation (Forane®, Baxter, UK). Representative tissue specimens from all animals’ intestines, livers, and spleens were carefully collected and fixed in a 10% neutral buffered formalin solution for 48 hours. Following fixation, the tissues were dehydrated through a graded series of ethyl alcohol concentrations, cleared in xylene, and subsequently embedded in paraffin wax. Two sections, each 5 µm thick, were obtained from each tissue specimen. The sections were then deparaffinized in xylene and rehydrated through a descending series of alcohol concentrations. After rehydration, the tissue sections were mounted onto histological slides and stained with hematoxylin and eosin (H&E) for microscopic examination. The prepared slides were thoroughly analyzed under a light microscope to identify pathological alterations and evaluate the administered drug’s potential ameliorative effects. This standardized protocol ensured accurate and consistent histological assessment across all samples [39].

**Histopathological scores for intestine:** H&E-stained tissue sections of the intestine were evaluated in a blinded manner by assessing specific histological parameters, including mononuclear cell infiltration in the lamina propria, crypt hyperplasia, depletion or hyperplasia of goblet cells, and alterations in mucosal histoarchitecture. A systematic scoring approach was employed to quantify these histological changes. Histological alterations were counted in 50 randomly selected microscopic fields (captured images; 40 × objective) from each mouse within each experimental group. This method ensured a comprehensive and unbiased assessment of intestinal pathology across all samples [40].

**Immunohistochemical examination for detecting intestinal dysplasia using cyclin D1 marker:** Three to five-micrometer intestinal tissue sections were deparaffinized using xylene and rehydrated using varying degrees of alcohol. Next, peroxidase activity was blocked using 3% hydrogen peroxide. The Dako target retrieval solution (pH 6.0) was applied for twenty minutes. The sections were treated for one hour with the primary antibody (Anti-Cyclin D1 antibody [SP4], ab 16663, Abcam, UK, diluted 1/100 in PBS). Antibody binding was identified using Dako’s HRP Envision Kit (Dako Cytomation, Denmark) [41].

**Immunological measurements:** Interleukin-10 (IL-10) and interferon-gamma (IFN-γ) levels in mouse sera were measured. Blood samples were collected in plain tubes from the mice’s retroorbital veins at the indicated time points (14 dpi and 21 dpi). After 30 minutes at room temperature and 15 minutes of centrifugation at 3000 rpm, sera were collected and stored at −20°C. Using an ELISA kit and following the manufacturer’s instructions, the enzyme-linked immunosorbent assay (ELISA) was employed to measure the levels of IFN-γ and IL-10 in the serum (Bioneovan Co.; Ltd, Beijing, China).

### Pharmacokinetic study

The pharmacokinetics of THQ, THQ/CsNPs, NTZ, and NTZ/CsNPs were evaluated in Wistar albino rats (220−250 g) following a previously reported method with slight modifications [42]. Before the study, the animals were fasted overnight and randomly divided into four groups, each consisting of six rats: Group I: treated with THQ; Group II: treated with THQ/CsNPs; Group III: treated with NTZ; Group IV: treated with NTZ/CsNPs. All groups received an oral dose of 20 mg/kg of their respective formulations. Blood samples (500 μL) were collected from the ocular vein into heparinized tubes at intervals of 0.5, 1, 2, 4, 6, 8, 12, and 24 hours post-administration. The samples were centrifuged at 10,000 rpm for 5 min to isolate plasma stored at −20°C. Plasma samples were deproteinized by adding 1 mL of acetonitrile to each sample, followed by vortexing for 10 min and centrifugation at 6,000 rpm for another 10 min. The supernatant was collected, and the THQ and NTZ concentrations in plasma at various time points were measured using RP-HPLC. Pharmacokinetic parameters such as peak plasma concentration (*C*_max_), time to reach peak concentration (*T*_max_), mean residence time (MRT), area under the curve (AUC), elimination rate constant (*K*_el_), and plasma half-life (*t*_1/2_) were calculated using PK Solver 2.0 software through non-compartmental analysis.

### Statistical analysis

Data analysis was conducted using IBM SPSS Statistics for Windows, version 26. Continuous normally distributed variables were expressed as mean ± SE. Multi-group comparisons were made using Tukey HSD’s post hoc analysis after one-way ANOVA. A p-value was considered highly significant if less than 0.001 and statistically significant if less than 0.05.

## Results

### X-ray diffraction (XRD) pattern

**Fig 1:**
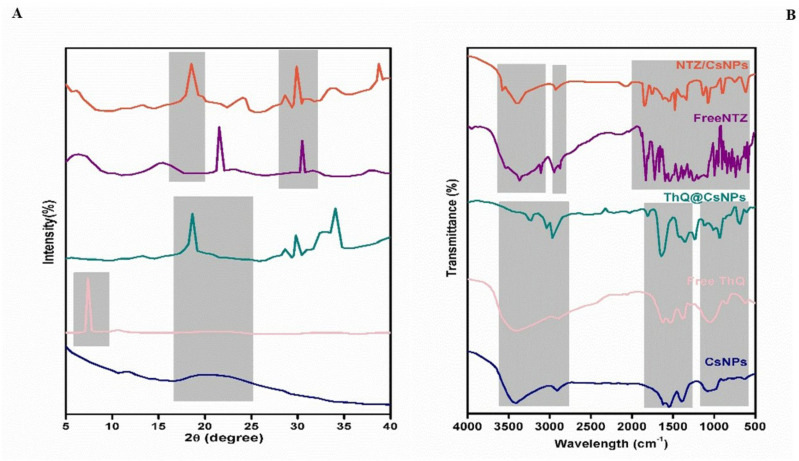
(A) XRD and (B) FTIR of CsNPs, free THQ, THQ/CsNPs, free NTZ, and NTZ/CsNPs.

XRD analysis was conducted on CsNPs, pure THQ, pure NTZ, THQ/CsNPs, and NTZ/CsNPs, as shown in Fig 1A. The XRD pattern of CsNPs exhibited a broad diffuse peak centered at around 20°, indicating the amorphous nature of chitosan with disordered chain arrangements in the nanoparticles. High-resolution X-ray powder diffraction characterized the crystal structure of THQ at the nanoscale and provided insights into its chemical behavior. Compared to published XRD data on bulk THQ crystals, THQ, with the chemical formula 2-isopropyl-5-methyl-1,4-benzoquinone, has been previously shown to exhibit good crystallinity in its bulk form using Rietveld refinement analysis of XRD data [43]. In this study, the XRD pattern of THQ displayed a peak at 8.4997° 2θ with a relative intensity of 100% and d = 10.40313 Å. The XRD pattern of NTZ displayed the strongest peak at 5.332° 2θ with d = 16.59 Å.

The XRD patterns of the THQ/CsNPs and NTZ/CsNPs were compared to empty CsNPs, as shown in Fig 1A. The broad diffraction peak of THQ/CsNPs and the strong peak of NTZ/CsNPs were slightly shifted to higher angles compared to CsNPs. Peak shifts can arise from changes in interatomic distances and crystal lattice parameters caused by doping, temperature effects, or strain. The observed shift suggests that the encapsulation of THQ and NTZ altered the CsNPs structure, leading to variations in interplanar spacings. THQ and NTZ loading potentially changed the interaction angles and atomic arrangements. Surface adsorption or entrapment of THQ and NTZ within the Cs matrix could induce such effects by disrupting the original Cs structure. This indicates that crystalline THQ and NTZ were converted to amorphous when dispersed into the polymeric nanoparticles. Overall, the changes in the XRD pattern, including peak position, shape, and relative intensity, confirm the loading of THQ and NTZ into the CsNPs, resulting in structural changes detectable by diffraction analysis.

### FTIR spectral studies

As illustrated in Fig 1B, the FTIR spectrum of CsNPs displayed characteristic peaks consistent with the structure of the chitosan polymer. A broad peak was observed at 3419 cm^-1^, corresponding to overlapping O-H and N-H stretching vibrations. Other notable peaks included aliphatic C-H stretches at 2900 cm^-1^, N-H in-plane bending at 1662 cm^-1^, C-O stretches of primary alcohols at 1414 cm^-1^, and C3-OH hydroxyl stretches at 1098 cm^-1^ [44]. The FTIR spectrum of THQ displayed several characteristic peaks corresponding to key functional groups in its chemical structure, including C-H, -CH_2_, -CH_3_, C = O, C-O, and C = C bonds. At 1651 cm^-1^, a distinct strong stretching peak was detected and attributed to the carbonyl C = O bond of the cyclohexadiene ring in THQ. An intense band at 2967 cm^-1^ represents aliphatic C-H stretching. A weak C = C stretch around 3180 cm^-1^ corresponds to the vinyl group. An overlapping strong carboxylic C = O stretch makes unambiguous identification of a weak C-C stretch difficult in this region [45].

NTZ’s FTIR spectra showed distinctive absorption bands at 1771 cm^-1^ and 1617 cm^-1^, which are associated with the carbonyl (C = O) groups of the ester and amide links in its molecule [46]. The amide carbonyl group is represented by the band at 1671 cm^-1^. The broadband corresponding to hydroxyl (-OH) groups was seen around 3256−2578 cm^-1^.

The FTIR spectrum of THQ/CsNPs and NTZ/CsNPs showed distinct differences in peak patterns and positions compared to the spectra of the individual components CsNPs, THQ, and NTZ. The changes in the FTIR peaks of THQ/CsNPs and NTZ/CsNPs indicate interactions between the CsNPs and THQ molecules, confirming the successful loading of THQ and NTZ into the chitosan nanoparticles. The spectral variations, including shifts in absorptions and intensity changes, are attributable to interactions arising from the encapsulation of THQ within the chitosan nanoparticle matrix.

### Hydrodynamic size, PDI, and ζ–potential

**Fig 2:**
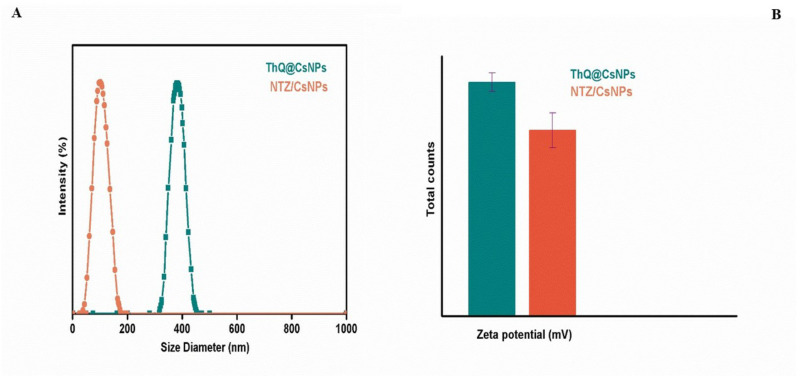
(A) Particle size analysis and (B) Zeta potential of THQ/CsNPs and NTZ/CsNPs.

The hydrodynamic size and PDI of the THQ/CsNPs and NTZ/CsNPs were measured using DLS. DLS determines particle size by measuring diffusion coefficients derived from light scattering fluctuations caused by the Brownian motion of nanoparticles in suspension. The Z-average diameter obtained represents the mean hydrodynamic size and is considered the most reliable parameter for quality control purposes, as defined in ISO standards. For THQ/CsNPs, the Z-average size was 380.1 ± 8 nm, and the PDI was 0.312, as shown in Fig 2A and 2B. The PDI indicates a relatively wide size distribution. For NTZ/CsNPs, the Z-average size was 103.15 ± 5 nm, and the PDI was 0.201. The zeta potential was + 40.5 ± 1.6 mV for THQ/CsNPs and +32.2 ± 3 mV for NTZ/CsNPs, suggesting high surface charge and colloidal stability of the nanoparticles. Values above +30 mV or below −30 mV typically confer sufficient repulsive forces for preventing aggregation [47].

### Surface morphology (SEM)

**Fig 3:**
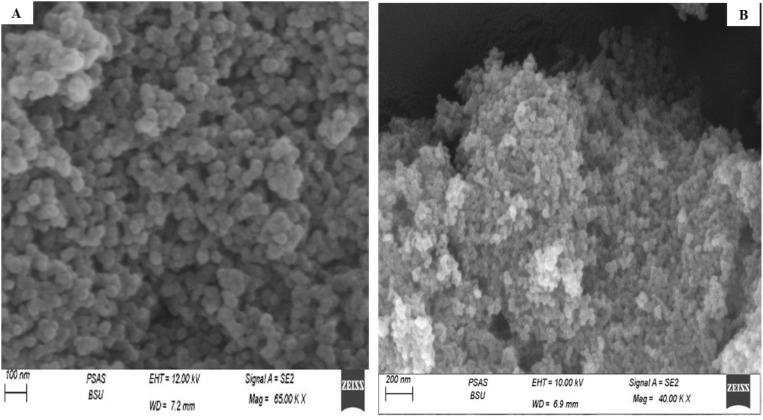
SEM photographs of (A) THQ/CsNPs and (B) NTZ/CsNPs.

SEM was utilized to investigate the surface texture and shape of the optimized THQ/CsNPs (Fig 3A) and NTZ/CsNPs (Fig 3B). As shown in Fig 3, the SEM analysis of both prepared nanocomposites demonstrated that the nanoparticles had a smooth, spherical geometry and did not clump or adhere to each other, suggesting that the formulations were stable. The individual particulate nature, without agglomeration, observed under SEM confirms the successful synthesis of discrete, stable nanoparticles.

### Entrapment efficiency and loading capacity

The entrapment efficiency and loading capacity of THQ drug in THQ/CsNPs and NTZ drug in NTZ/CsNPs were determined by spectrophotometric analysis at 257 and 222 nm, respectively. The entrapment efficiency of THQ in THQ/CsNPs was found to be 84.21 ± 0.46%, and the loading capacity was 17.11 ± 0.14%. For NTZ/CsNPs, the entrapment efficiency and loading capacity were 92.39 ± 0.21% and 12.31 ± 0.15%, respectively. The high entrapment efficiency percentage indicates effective encapsulation of both THQ and NTZ into the Cs NPs. This influences drug release kinetics and overall formulation efficacy. Encapsulation in the Cs NPs helps protect the loaded THQ and NTZ while providing biocompatible and biodegradable carrier properties.

### In vitro release profile

**Fig 4:**
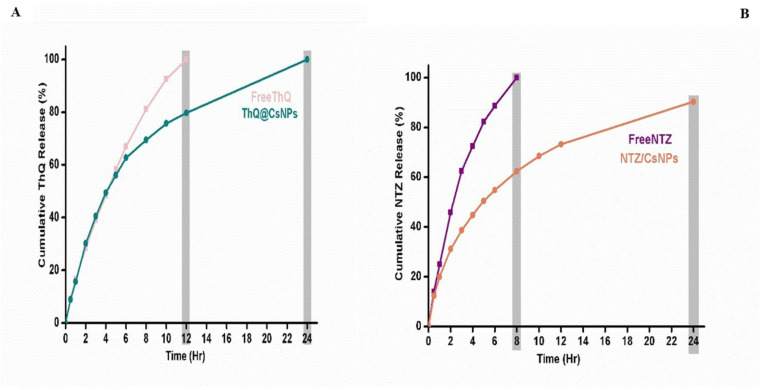
In vitro release profile of (A) free THQ and THQ/CsNPs and (B) free NTZ and NTZ/CsNPs.

The in vitro release profiles of free THQ and free NTZ loaded in THQ/CsNPs and NTZ/CsNPs were evaluated in PBS (pH 6.8) at 37 ± 5°C for 24 hours, as shown in Fig 4. THQ and NTZ exhibited a biphasic release pattern from CsNPs, characterized by an initial rapid burst release followed by a slower sustained release phase up to 24 hours. Free THQ showed 100% rapid release after 12 hours, while THQ/CsNPs displayed a rapid release of 56% in the first 5 hours, with a lower burst release reaching 100% THQ by 24 hours (Fig 4A). The slower release rate of THQ from CsNPs could be attributed to the entrapment of THQ within the nanoparticle matrix, compared to the immediate release of the free drug. The release profiles of free NTZ and NTZ/CsNPs showed a rapid release of free drug (100%) in the first eight hours, compared to only 81% released in the same eight-hour period under similar conditions (Fig 4B). Subsequently, NTZ/CsNPs demonstrated a sustained-release characteristic, with the cumulative drug release at 24 hours reaching about 100%. The initial burst may correspond to free THQ and NTZ localized on the nanoparticle surface, while the later sustained release could arise from the diffusion of both drugs from the core as chitosan swells (Fig 4).

### Cost analysis of CsNPs, THQ/CsNPs, and NTZ/CsNPs

Cost estimation is an essential factor in assessing the feasibility of nanoparticle formulations for real-world applications. The cost analysis of THQ/CsNPs and NTZ/CsNPs revealed that THQ/CsNPs are more cost-effective than NTZ/CsNPs. The total production cost for THQ/CsNPs was $0.074 USD/g, while NTZ/CsNPs had a slightly higher cost of $0.077 USD/g. This difference is attributed to the optimized synthesis process for THQ/CsNPs, which reduced the amount of thymoquinone used, minimized energy consumption during stirring and ultrasonication, and utilized bulk-purchased raw materials to lower overall costs. Both formulations demonstrated economic feasibility for large-scale production, with THQ/CsNPs offering a slight cost advantage over NTZ/CsNPs. The results are summarized in Tables 1 and 2.

**Table 1:** Cost estimation details of NTZ/CsNPs.

Material	Purchased Quantity (g)	Total Purchase Cost (USD)	Purchasing Cost (USD/g)	Used Quantity (g or mL)	Cost of used quantity (USD)
Chitosan	500	$50	$0.10	0.3	$0.03
Acetic Acid	1000	$10	$0.01	1	$0.01
Tripolyphosphate (TPP)	100	$25	$0.25	0.1	$0.03
Nitazoxanide (NTZ)	100	$55	$0.55	0.05	$0.03
DMSO	1000	$30	$0.03	5	$0.15
Equipment	**Time (h)**	**Max Power (kW)**	**Unit Cost of Power (USD/kWh)**	**Energy Cost (USD)**	
Magnetic Stirrer	2	1	$0.16	$0.32	
Ultrasonicator	1	1	$0.16	$0.16	
yield				10	g
Total Cost				$0.77	USD
Cost per gram:				$0.077	USD/g

**Table 2:** Cost estimation details of THQ/CsNPs.

Material	Purchased Quantity (g)	Total Purchase Cost (USD)	Purchasing Cost (USD/g)	Used Quantity (g or mL)	Cost of used quantity (USD)
Chitosan	500	$50	$0.1	0.3	$0.03
Acetic Acid	1000	$10	$0.01	1	$0.01
Tripolyphosphate (TPP)	100	$25	$0.25	0.1	$0.025
Thymoquinone (THQ)	100	$50	0$.5	0.05	$0.025
DMSO	1000	$30	$0.03	5	$0.15
Equipment	**Time (h)**	**Max Power (kW)**	**Unit Cost of Power (USD/kWh)**	**Energy Cost (USD)**	
Magnetic Stirrer	2	1	$0.16	$0.32	
Ultrasonicator	1	1	$0.16	$0.16	
yield				10	g
Total Cost				$0.74	USD
Cost per gram:				$0.074	USD/g

### MTT assay

**Fig 5:**
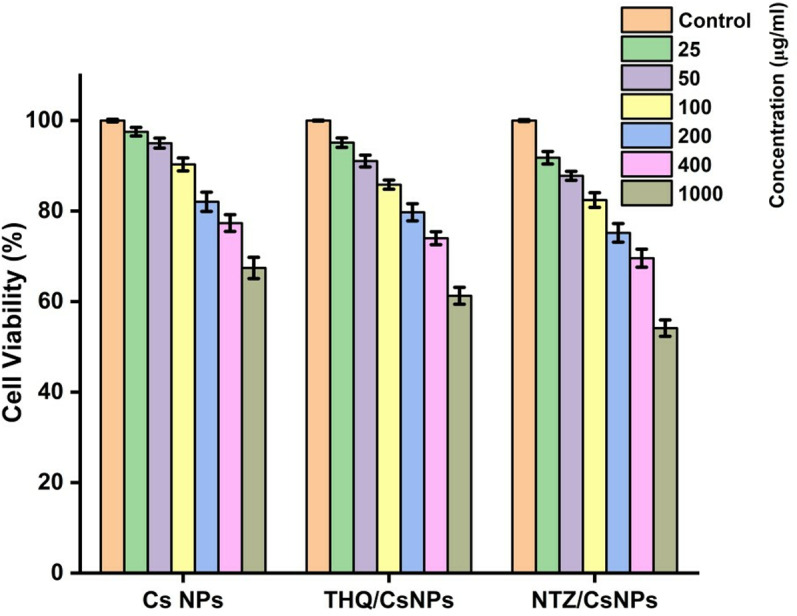
Cytotoxicity of Cs NPs, THQ/CsNPs, and NTZ/CsNPs on HT-29 cells at various concentrations. The percentage of cell viability is shown as a function of nanoparticle concentration (100 µg/mL to 0.0125 µg/mL). Data are presented as mean ± standard deviation (n = 3).

The cytotoxicity of Cs NPs, THQ/CsNPs, and NTZ/CsNPs was evaluated on HT-29 cells at various concentrations (Fig 5). The results demonstrated a dose-dependent effect on cell viability for all formulations. Cs NPs showed minimal cytotoxicity, with cell viability at 97.54% ± 0.96% at the highest concentration (1000 µg/mL) and decreasing to 67.44% ± 2.35% at the lowest concentration (25 µg/mL). THQ/CsNPs exhibited slightly higher cytotoxicity compared to Cs NPs, with cell viability ranging from 95.12% ± 1.05% at 1000 µg/mL to 61.29% ± 1.86% at 25 µg/mL. NTZ/CsNPs demonstrated the highest cytotoxicity among the three formulations, with cell viability decreasing from 91.79% ± 1.38% at 1000 µg/mL to 54.13% ± 1.79% at 25 µg/mL. These findings indicate that the nanoparticles’ cytotoxic effects depend on their concentration and payload.

### Analysis of the oocyst shedding

**Fig 6:**
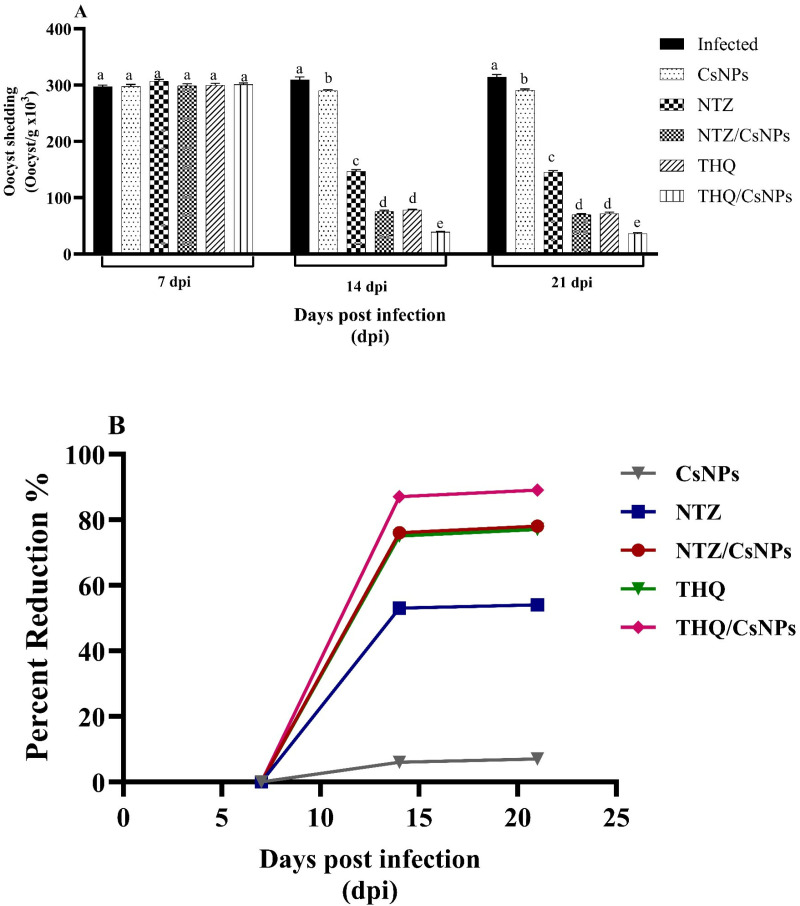
(A) Oocyst shedding in different study groups at 7, 14, and 21 dpi. Results are displayed as mean ± SE. Different letters indicate significant differences, while mean values with the same letters are similar. (B) Percent reduction of the oocysts in the different study groups at 7, 14, and 21 dpi.

The diseased model group (GII) had the highest mean count of *Cryptosporidium* oocysts on the 21^st^ dpi, corresponding to the end of the experiment (314.4 ± 4.4). The infected mice treated with CsNPs (GIII) had the second-highest mean count (291.1 ± 2), with a statistically significant difference between the two groups (P < 0.001). The treated groups (GVI-V) had a significantly reduced mean count of *Cryptosporidium* oocysts compared to the infected non-treated group (GII) (P < 0.001). Comparing the effectiveness of nitazoxanide (54%) and thymoquinone (77%) revealed a highly significant difference (P < 0.001). The effectiveness of nitazoxanide loaded onto CsNPs (78%) and thymoquinone alone (77%) revealed no significant difference (P = 0.9). When thymoquinone and nitazoxanide were loaded onto CsNPs, their respective effectiveness increased to 89% and 78%, with a highly significant difference (P < 0.001) (Table 3 and Fig 6A, B).

**Table 3:** Count of *Cryptosporidium* oocysts per gram stool (x10^3^) in different experimental groups.

Groups	7^th^ day PI	14^th^ day PI		21^th^ day PI	
	Mean ± SE	Mean ± SE	I %	Mean ± SE	I%
GII (Infected)	297.8 ± 2.4^Aa^	309.8 ± 4.6 ^Aa^		314.4 ± 4.4 ^Aa^	
GIII (CsNPs)	297.8 ± 3.5 ^Aa^	290.2 ± 1.7 ^Ab^	6%	291.1 ± 2^Ab^	7%
GIV(NTZ)	307.3 ± 3 ^Aa^	146.8 ± 2.9 ^Bc^	53%	145.5 ± 2.6 ^Bc^	54%
GV (NTZ/CsNPs)	298.7 ± 3.8 ^Aa^	75.9 ± 1.8 ^Bd^	76%	70.1 ± 1.3 ^Cd^	78%
GVI (THQ)	300 ± 3.1 ^Aa^	78 ± 1.5 ^Bd^	75%	72 ± 2.6 ^Cd^	77%
GVII (THQ/CsNPs)	301 ± 2.9 ^Aa^	39.2 ± 1.2 ^Be^	87%	36.1 ± 1.8 ^Be^	89%

Table 3 displays the number of oocysts in gram feces as (mean ± SE) × 10^3^. The following formula was used to determine the percentage of inhibition:

%Inhibition = [(mean oocyst count in the infected untreated group − mean oocyst count in the infected treated group)/mean oocyst count in the infected untreated group] × 100

In each row, different superscript capital letters on the mean values indicate significant differences, while mean values with the same letters are similar. In each column, different superscript small letters on the mean values indicate significant differences, while mean values with the same letters are similar. PI denotes post-infection, and I% denotes percent inhibition.

### Histopathological results

#### Intestine.

**Fig 7:**
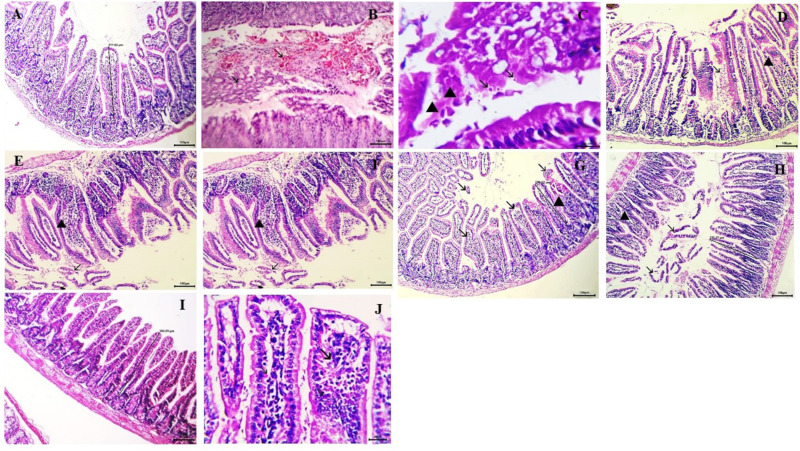
Histopathological findings of small intestine sections (H&E staining). A: Healthy control group showing normal villous architecture and normal brush border (X100). B and C: Infected control group. B: Showing excess mucus admixed with RBCs, epithelial sheets, inflammatory cells, and *Cryptosporidium* oocysts within the intestinal lumen (arrow) (X400). C: Showing desquamated mucosal epithelial cells (arrowhead) and invasion of *Cryptosporidium* oocysts within the intestinal lumen (arrow) (X1000). D: GIII treated with chitosan showing severe destruction of villi with exposure of lamina propria (arrow) and heavy infiltration of inflammatory cells (arrowhead) (X100). E: GIV treated with NTZ showing enteritis, represented by desquamation of epithelium (arrow), thickened villi with infiltration of inflammatory cells, and submucosal edema (arrowhead). F: GV treated with NTZ-loaded chitosan nanoparticles showing hyperplasia of goblet cells, mild inflammatory cell infiltration (arrowhead), and normal villi (arrow) (X100). G: GVI treated with thymoquinone showing moderate inflammatory cell infiltration in the mucosa (arrowhead) and moderate desquamation of epithelium (arrow) (X100). H and I: GVII treated with thymoquinone-loaded chitosan nanoparticles. H: Showing normal mucosa, goblet cells, and restoration of the normal villous pattern with no inflammatory cells (arrow) (X100). I: Showing regenerative attempts of enterocytes (re-epithelialization) (arrow) (X400).

The immunosuppressed group (GI) exhibited normal intestinal architecture, with average villi width and length, a moderate number of goblet cells, and a well-defined brush border (Fig 7A). In contrast, the control infected, non-treated group (GII) displayed pronounced histopathological alterations, including abnormal villous architecture (stunted villi), marked inflammatory cell infiltration, mucosal ulceration, excess mucus mixed with red blood cells (RBCs), epithelial sheets, oocysts in the lumen, submucosal edema, and dilated lymphatic vessels. Additionally, depletion of gut-associated lymphoid tissue (GALT) and significant activation of Paneth cells were observed. *Cryptosporidium* oocysts, appearing as purple-stained round to oval bodies measuring 4–6 µm, were detected along the brush border of intestinal villi and within crypts (Fig 7B, C).

Group III, treated with chitosan nanoparticles alone, exhibited similar histopathological changes without significant improvement in intestinal lesions (Fig 7D). Group IV, treated with NTZ, showed persistent villous atrophy and mild improvement in histopathological alterations, including inflammatory cell infiltration in the lamina propria and the presence of mucosal oocysts (Fig 7E). In contrast, the NTZ-loaded chitosan nanoparticles-treated group (GV) demonstrated better improvement, with only mild inflammatory cells in the mucosa and submucosa and the absence of oocysts (Fig 7F).

Group VI, treated with thymoquinone alone, exhibited greater improvement than the NTZ-treated group, although some histopathological changes persisted (Fig 7G). The most significant improvement was in Group VII (GVII), treated with thymoquinone-loaded chitosan nanoparticles. This group displayed restored villous, mucosal, and submucosal histomorphology, with no evidence of inflammatory cells or other pathological alterations (Fig 7H, I). These findings highlight the superior therapeutic efficacy of thymoquinone-loaded chitosan nanoparticles in ameliorating *Cryptosporidium*-induced intestinal damage. Statistically, the mean ± SE values for the investigated lesions are presented in Table 4.

**Table 4:** Histopathological scores for the intestine.

Lesion frequency/morphometric indices		GI (negative control)	GII (infected)	GIII (CsNPs)	GIV (NTZ)	GV (NTZ/CsNPs)	GVI (THQ)	GVII (THQ/CsNPs)
**Lesion frequency**	Epithelial necrosis	0 ± 0^C^	18.33 ± 0.42^A^	18 ± 0.58^A^	9 ± 0.58^B^	0.5 ± 0.22^C^	7.5 ± 0.43^B^	0 ± 0^C^
	Goblet cell hyperplasia	18 ± 0.37^C^	37.83 ± 0.7^A^	37.5 ± 0.92^A^	21.5 ± 0.76^B^	13.33 ± 0.49^D^	20.67 ± 0.49^BC^	11.5 ± 0.62^D^
	Mucosal-associated lymphocyte hyperplasia	9 ± 0.37^E^	13 ± 0.73^D^	13 ± 0.68^D^	21 ± 0.58^C^	30 ± 0.68^B^	22 ± 0.68^C^	37 ± 0.89^A^
	Vascular congestion	0 ± 0^D^	31.33 ± 2.42^A^	30.67 ± 1.71^A^	21 ± 0.97^B^	5.67 ± 0.67^C^	18.5 ± 0.85^B^	0 ± 0^D^
	Mucosal erosion	0 ± 0^D^	31.83 ± 1.08^A^	30 ± 1.06^A^	16 ± 0.58^B^	5.67 ± 0.67^C^	14.83 ± 0.65^B^	0 ± 0^D^
	Mucosal ulceration	0 ± 0^C^	20.67 ± 1.02^A^	21.17 ± 1.35^A^	9.83 ± 0.6^B^	0 ± 0^C^	8.83 ± 0.48^B^	0 ± 0^C^
	Villus atrophy	0 ± 0^C^	26.5 ± 0.92^A^	24.83 ± 1.35^A^	10 ± 0.58^B^	0.33 ± 0.21^C^	9.83 ± 0.87^B^	0 ± 0^C^
	Villus fusion	0 ± 0^C^	11 ± 0.45^A^	11 ± 0.73^A^	5.17 ± 0.31^B^	0.33 ± 0.21^C^	4.83 ± 0.17^B^	0 ± 0^C^
**Morpho metric indices**	Villus height	392.17 ± 2.46^A^	274.33 ± 3.07^D^	275.83 ± 2.5^D^	298.67 ± 2.4^C^	370 ± 5.32^B^	302 ± 2.35^C^	391.5 ± 2.09^A^
	Crypt depth	78.15 ± 0.21^C^	80.25 ± 0.24^A^	80.1 ± 0.22^A^	79.7 ± 0.21^AB^	78.82 ± 0.34^BC^	79.7 ± 0.22^AB^	78.4 ± 0.34^C^
	Villus width	97.5^A^	90.2^A^	87.3^A^	91.2^A^	94.3^A^	91.4^A^	97.6^A^
	Villus surface area	38038.33 ± 203.44^A^	24354.45 ± 349^D^	24282.1 ± 328.08^D^	27609.37 ± 307.03^C^	35264.92 ± 567.74^B^	27975.53 ± 357.01^C^	38039.2 ± 179.12^A^
	Thickness of lamina propria	159 ± 2.73^D^	193.33 ± 2.42^A^	191.83 ± 2.71^A^	174.33 ± 1.05^B^	160.33 ± 3.35 CD	170.33 ± 1.15^BC^	159.17 ± 2.91^D^
	Thickness of muscular layer per image	55.6 ± 0.65^D^	68.12 ± 0.31^AB^	68.28 ± 0.15^A^	65.87 ± 0.59^BC^	57.8 ± 0.72^D^	64.35 ± 0.2^C^	55.6 ± 0.65^D^

Values are represented as the mean ± SE. The means within the same row carrying different superscripts are significant at p < 0.05. Similar letters indicate no significant difference, while different letters indicate significant difference

#### Liver.

**Fig 8:**
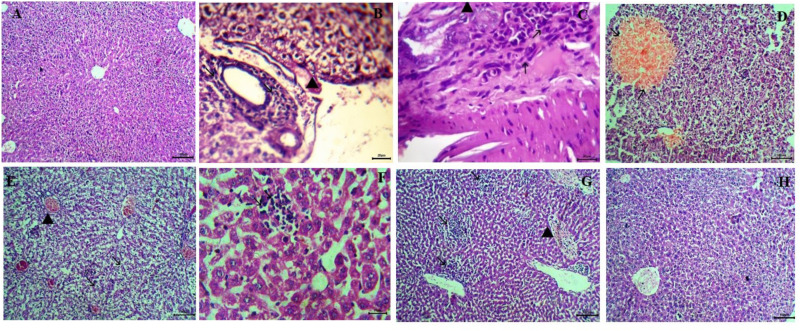
Histopathological findings of liver section (H&E staining). A: Showing normal liver architecture of healthy control group (X100). B and C: Infected control group. B: Showing portal mononuclear cell aggregates (arrow) and acute cell swelling of hepatocytes (arrowhead) (X100). C: Showing hepatic necrotic area replaced by mononuclear cells, fibroblasts (arrow), and giant cells (arrowhead) (X400). D: Mice treated with chitosan showing a focal area of coagulative necrosis (arrow) (X100). E: Mice treated with NTZ alone showing hydropic degeneration (arrow) and congestion of central vein (arrowhead) (X100). F: Mice treated with NTZ loaded on chitosan nanoparticles showing mild interstitial mononuclear cell aggregation (arrow) (X400). G: Mice treated with thymoquinone showing multiple aggregations of inflammatory cells (arrow) with congestion of central vein (arrowhead) (X100). H: Mice treated with thymoquinone loaded on chitosan nanoparticles showing normal histological architecture (X100).

Healthy mice (GI) showed normal liver architecture (Fig 8A). In contrast, the liver of infected non-treated mice (GII) exhibited mononuclear inflammatory cell aggregates in the portal vein, acute cell swelling, hyperplasia of Kupffer cells, focal necrosis, microsteatosis, and hepatocyte atrophy (Fig 8B, C). Mice treated with chitosan revealed similar changes as control-infected mice (Fig 8D). Mice treated with NTZ alone and thymoquinone alone showed partial improvement, with the presence of degenerative changes in hepatic parenchyma in both groups (Fig 8E, G). However, mice treated with NTZ loaded on chitosan nanoparticles showed moderate ameliorative effects. Still, they exhibited mild interstitial inflammatory cells and congestion (Fig 8F). Mice treated with thymoquinone loaded on chitosan nanoparticles showed marked improvement in liver histopathological changes, resembling normal histological patterns (Fig 8H).

#### Spleen.

**Fig 9:**
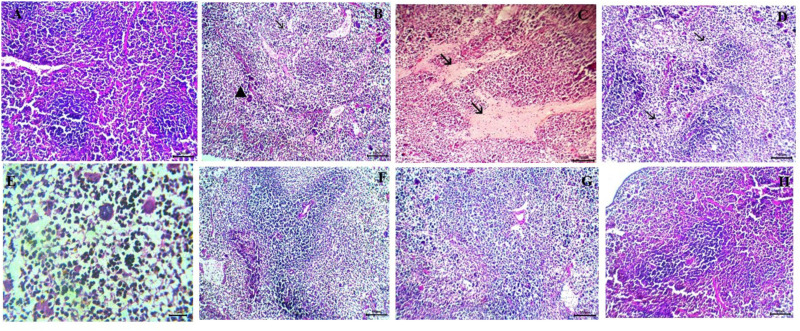
Histopathological findings of spleen sections (H&E staining). A: Healthy control group showing normal lymphoid follicle (X100). B-C: Control infected group. B: Showing severe depletion of the lymphoid follicle (arrow) with the presence of megakaryocytes (arrowhead) (X100). C: Showing inflammatory edema (arrow) (X400). D: Group treated with chitosan showing depleted white pulp (X100). E: Group treated with NTZ showing moderate lymphoid depletion with the presence of megakaryocytes (X400). F: Group treated with NTZ loaded on chitosan nanoparticles showing mild depletion of lymphoid follicle (X100). G: Group treated with thymoquinone showing moderate lymphoid follicle depletion (X100). H: Group treated with thymoquinone loaded on chitosan nanoparticles showing intense lymphoid hyperplasia similar to normal (X100).

The spleen of the healthy group showed normal red and white pulp with supporting stroma (Fig 9A). In contrast, both the infected control group (Fig 9B, C) and the group treated with chitosan (Fig 9D) exhibited severe depletion of lymphoid elements with extramedullary megakaryocytes and inflammatory edema within splenic tissue. Both groups treated with NTZ (Fig 9E) and THQ (Fig 9G) showed partial improvement with mild hyperplasia of lymphoid elements. The group treated with NTZ loaded on chitosan nanoparticles demonstrated much more improvement in restoring lymphoid content (Fig 9F). However, the best improvement was observed in the group treated with thymoquinone loaded on chitosan nanoparticles, which showed histological features similar to normal splenic tissue with marked hyperplasia of lymphocytes (Fig 9H).

### Immunohistochemical staining of cyclin D1

**Fig 10:**
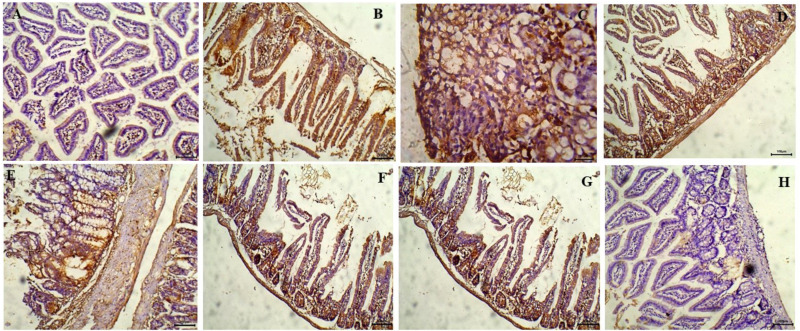
Representative photomicrographs of immunoreactivity for cyclin D1 in small intestine sections (H&E staining). A: Healthy control group showing negative immunoreactivity (X100). B, C: Infected control group. B: Showing strong immunoreactivity, manifested by brown granules in the intestinal mucosa and crypts (X100). C: Showing the dysplastic nuclei with strong immunoreactivity (X400). D: Group treated with chitosan showing strong immunoreactivity (X100). E-H: Treated groups demonstrated reductions in cyclin D1 expression.

A negative cyclin D1 immunoreactivity was observed in the small intestine of healthy control mice (Fig 10A). Both the control-infected group (Fig 10B, C) and the chitosan-treated group exhibited intense immunostaining (Fig 10D), characterized by dense brown granules in dysplastic cells of the intestinal mucosa and crypts. Moderate immunostaining for cyclin D1 was observed in the intestinal section of mice treated with NTZ (Fig 10E) and thymoquinone (Fig 10G). A weak immunostaining was detected in the intestine of mice treated with NTZ-loaded chitosan nanoparticles (Fig 10F), while the intestine of mice treated with thymoquinone-loaded chitosan nanoparticles showed no immune deposits for cyclin D1 (Fig 10H).

### Immunological analysis of serum IFN-γ and IL-10

**Fig 11:**
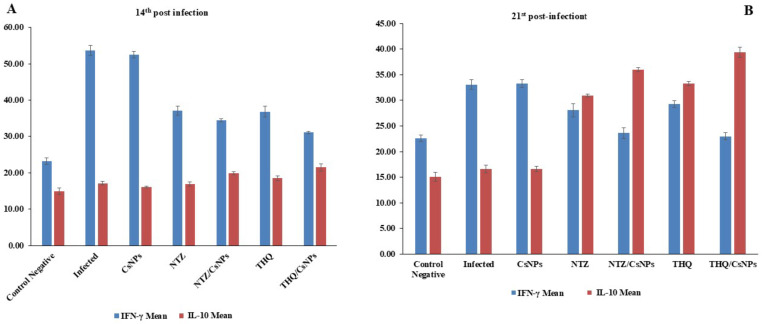
A: Serum levels of interferon-gamma (IFN-γ) and interleukin-10 (IL-10) among the experimental groups at 14^th^ post-infection. B: Serum levels of interferon-gamma (IFN-γ) and interleukin-10 (IL-10) among the experimental groups at 21^st^ post-infection. Results are displayed as mean ± SE.

One week after drug administration (14 dpi), the one-way ANOVA revealed significant differences in serum levels of IFN-γ and IL-10 among the experimental groups (Table 5). Compared to the control negative group, the control positive group exhibited significantly elevated IFN-γ levels (p < 0.001). While all treatment groups showed lower IFN-γ levels than the control positive group, the THQ/CsNPs group (GVII) had the lowest levels, significantly different from the control positive group (p < 0.001). IL-10 levels were significantly elevated in all groups compared to the control negative group (p < 0.001). The highest levels were observed in the THQ/CsNPs group, followed by the NTZ/CsNPs group. These groups showed significantly higher IL-10 levels than the control positive group (p < 0.01) (Table 5 & Fig 11A).

**Table 5:** Levels of IFN-γ and IL-10 at 14 days post-infection.

14^th^ post-infection				
**Groups**	**IFN-γ**		**IL-10**	
	**Mean ± SE**	**ANOVA** ***P*. value**	**Mean ± SE**	**ANOVA** ***P*. value**
**GI (Control Negative)**	23.2 ± 0.9 ^d^	0.001**	14.9 ± 0.9^d^	0.001**
**GII (Infected)**	53.7 ± 1.4 ^a^		17.1 ± 0.5^bd^	
**GIII (CsNPs)**	52.5 ± 0.9 ^a^		16.1 ± 0.3 cd	
**GIV(NTZ)**	37.1 ± 1.2 ^b^		16.9 ± 0.6 cd	
**GV (NTZ/CsNPs)**	34.4 ± 0.4 ^bc^		19.9 ± 0.4^ab^	
**GVI (THQ)**	36.8 ± 1.5 ^b^		18.5 ± 0.6^bc^	
**GVII (THQ/CsNPs)**	31.1 ± 0.3 ^c^		21.5 ± 1^a^	

P-value < 0.01 is highly significant.

At 21 days post-infection, significant differences in IFN-γ and IL-10 levels persisted among the groups (Table 5). The control positive group maintained significantly higher IFN-γ levels than the other groups (p < 0.001). The NTZ/CsNPs and THQ/CsNPs groups exhibited the lowest IFN-γ levels, significantly different from the control positive group (p < 0.001). IL-10 levels continued to be elevated in all groups compared to the control negative group. The THQ/CsNPs group maintained the highest IL-10 levels, followed by the NTZ/CsNPs group. These groups showed significantly higher IL-10 levels than the control positive group (Table 6 & Fig 11B).

**Table 6:** Levels of IFN-γ and IL-10 at 21 days post-infection.

21^st^ post-infection				
**Groups**	**IFN-γ**		**IL-10**	
	**Mean ± SE**	**ANOVA P. value**	**Mean ± SE**	**ANOVA *P*. value**
**C (Control Negative)**	22.6 ± 0.7 ^c^	0.001**	15.1 ± 0.9 ^d^	0.001**
**GII (Infected)**	33.1 ± 0.9 ^a^		16.6 ± 0.8 ^d^	
**GIII (CsNPs)**	33.3 ± 0.8 ^a^		16.6 ± 0.5 ^d^	
**GIV(NTZ)**	28.1 ± 1.3 ^b^		30.9 ± 0.3 ^c^	
**GV (NTZ/CsNPs)**	23.6 ± 1 ^c^		36 ± 0.4 ^b^	
**GVI (THQ)**	29.3 ± 0.6 ^ab^		33.3 ± 0.4 ^bc^	
**GVII (THQ/CsNPs)**	23 ± 0.7 ^c^		39.4 ± 1 ^a^	

P-value < 0.01 is highly significant.

### Pharmacokinetic study

**Fig 12:**
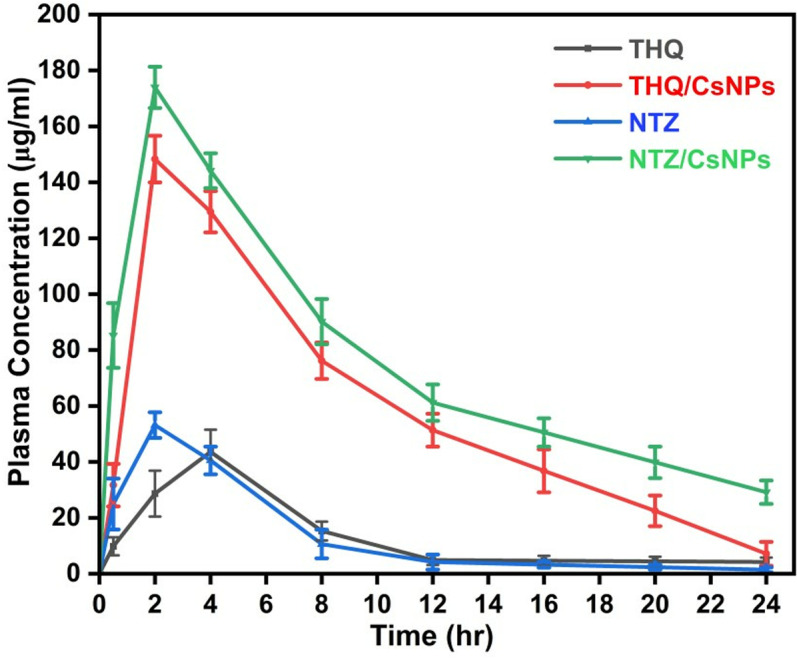
Comparative pharmacokinetic study of THQ, THQ/CsNPs, NTZ, and NTZ/CsNPs after oral administrations into rats (n = 6).

The pharmacokinetic study revealed distinct differences in the absorption and elimination profiles of THQ, THQ/CsNPs, NTZ, and NTZ/CsNPs, as shown in Table 7 and Fig 12. At 0 hour, no detectable drug concentrations were observed for all formulations. By 0.5 hours, composite formulations showed significantly higher plasma concentrations compared to their free drug counterparts, with THQ/CsNPs reaching 31.69 ± 7.61 µg/mL and NTZ/CsNPs achieving 85.21 ± 11.59 µg/mL, compared to free THQ (9.79 ± 3.25 µg/mL) and free NTZ (24.89 ± 9.11 µg/mL). The peak plasma concentration (*C*_max_) for free THQ was observed at four hours (43.6 ± 7.92 µg/mL), while THQ/CsNPs reached a much higher *C*_max_ of 148.28 ± 8.22 µg/mL at an earlier time point (two hours). Similarly, free NTZ exhibited a *C*_max_ of 53.15 ± 4.95 µg/mL at two hours, whereas NTZ/CsNPs achieved a significantly higher *C*_max_ of 173.94 ± 7.37 µg/mL at the same time. At 12 hours, THQ/CsNPs had a plasma concentration of 51.32 ± 5.9 µg/mL compared to free THQ at only 4.88 ± 1.79 µg/mL, and NTZ/CsNPs showed a concentration of 61.18 ± 6.49 µg/mL compared to free NTZ at just 4.16 ± 2.71 µg/mL. By the end of the study (24 hours), plasma concentrations for all formulations decreased significantly; however, composite formulations still retained measurable levels: THQ/CsNPs had a concentration of 7.11 ± 4.27 µg/mL compared to free THQ at 4.19 ± 1.58 µg/mL, while NTZ/CsNPs showed a concentration of 29.16 ± 4.2 µg/mL compared to free NTZ at only 1.39 ± 0.89 µg/mL.

**Table 7:** Pharmacokinetics parameters for THQ, THQ/CsNPs, NTZ, and NTZ/CsNPs after oral administrations into rats (n = 6).

Parameter	THQ	THQ/CsNPs	NTZ	NTZ/CsNPs
**AUC (µg•h/mL)**	340.37 ± 13.55	80.54 ± 2.73^a^	1438.07 ± 49.39	156.81 ± 2.41^a^
**AUC**₀** → 24 (µg•h/mL)**	315.97 ± 12.48	78.64 ± 5.32^a^	1440.72 ± 48.67	155.29 ± 6.21^a^
**AUC**₀**→∞ (µg•h/mL)**	629.6 ± 22.14	224.42 ± 10.87^a^	1465.46 ± 52.34	223.26 ± 8.19^a^
**MRT (h)**	6.97 ± 1.32	8.1 ± 0.98	7.97 ± 1.45	11.27 ± 1.12
***C***_**max**_ **(µg/mL)**	43.6 ± 7.92	148.28 ± 8.22^a^	53.15 ± 4.95	173.94 ± 7.37^a^
***T***_**max**_ **(h)**	4.0 ± 0.00	2.0 ± 0.00^a^	2.0 ± 0.00	2.0 ± 0.00
**Kel (h**˦** ¹)**	0.013 ± 0.0012	0.205 ± 0.0153^a^	0.105 ± 0.0087	0.0687 ± 0.006
***T*1/₂ (h)**	53.23 ± 4.88	3.37 ± 0.27^a^	6.55 ± 0.54	10.09 ± 0.89^a^

^a^Denotes highly significant (p < 0.05) values of THQ when compared with THQ/CsNPs and NTZ compared with NTZ/CsNPs.

## Discussion

The intracellular protozoan parasite *C. parvum* is one of the most prevalent worldwide and can cause intestinal disease in humans and animals [48]. It induces self-limited diarrhea in immunocompetent hosts and a life-threatening disease in immunocompromised hosts [49].

In the present study, mice were chemically immunosuppressed using dexamethasone. Due to its high glucocorticoid activity, it inhibits the immune system and the interferon-gamma pathway [50].

Finding a natural, non-toxic, and easily obtainable herbal substitute for the standard drugs on the market is a challenging task for researchers due to the adverse effects and increasing resistance of the antiparasitic drugs used for cryptosporidiosis treatment. Medicinal plants have played a significant role in producing potent therapeutic compounds for a very long time. Herbal remedies are in greater demand and have become increasingly popular [51].

The only anti-cryptosporidial medication approved by the US FDA is nitazoxanide. It facilitates the parasite’s removal by preventing its anaerobic metabolism for energy production [52]. However, nitazoxanide has a transient effect with relapses in immunocompetent individuals and is not completely effective in immunosuppressed patients without an effective host immune response [53]. The development of substitute medications to treat cryptosporidiosis remains necessary.

Significant antiparasitic efficacy has been discovered for thymoquinone (2-isopropyl-5-methyl-1,4-benzoquinone; TQ), the main pharmacologically active component of *Nigella sativa* (black seed) essential oil [54]. Drug delivery carriers in the nanoscale range can be created using nanotechnology, which helps to overcome the drawbacks of traditional drug delivery systems by being compact, target-specific, enhancing drug encapsulation, stable, and less toxic all at once [55]. Therefore, a novel treatment strategy for *C. parvum* infection could be using biosynthesized chitosan nanoparticles loaded with thymoquinone.

The current study was designed to investigate the potential therapeutic efficacy of thymoquinone alone or chitosan-loaded compared to the already prescribed medication nitazoxanide in an immunocompromised mouse model.

Regarding nano synthesis and characterization, in the current study, the absence of sharp peaks suggested decreased crystallite size and a lower degree of long-range order in the atomic/molecular structure of CsNPs compared to raw Cs powder [56]. The amorphous nature and lack of distinct crystalline planes are likely due to defects introduced in the nanoparticle nucleation and growth phases by the polymeric Cs chains. The shifts in peak positions for THQ/CsNPs and NTZ/CsNPs suggest that the encapsulation of THQ and NTZ alters the CsNPs structure. These shifts may arise from changes in interplanar spacings, possibly due to doping, temperature effects, or strain induced by the encapsulation process. The encapsulation of THQ and NTZ potentially changes the interaction angles and atomic arrangements within the CsNPs. Surface adsorption or entrapment of these compounds within the chitosan matrix could disrupt the original Cs structure, converting crystalline THQ and NTZ into an amorphous form when dispersed in the polymeric nanoparticles. Overall, the XRD pattern changes, including peak position, shape, and relative intensity, confirmed the successful loading of THQ and NTZ into the CsNPs [57]. These structural changes are detectable by diffraction analysis, highlighting the impact of encapsulation on the nanoparticle structure. This observation aligns with previous reports on chitosan nanoparticle formulations [58,59].

The FTIR spectrum of THQ/CsNPs and NTZ/CsNPs showed distinct differences in peak patterns and positions compared to the spectra of the individual components CsNPs, THQ, and NTZ. The changes in the FTIR peaks of THQ/CsNPs and NTZ/CsNPs indicated interactions between the CsNPs and THQ molecules, confirming the successful loading of THQ and NTZ into the chitosan nanoparticles. The spectral variations, including shifts in absorptions and intensity changes, are attributable to interactions arising from the encapsulation of THQ within the chitosan nanoparticle matrix [57,59,60].

The Z-average size and PDI values indicated differences in the size distribution of THQ/CsNPs and NTZ/CsNPs. THQ/CsNPs exhibited a larger average size and a broader size distribution, as indicated by the higher PDI, compared to NTZ/CsNPs. This suggests variations in the encapsulation process or particle formation mechanisms between the two types of nanoparticles. The zeta potential values for THQ/CsNPs and NTZ/CsNPs are above +30 mV, suggesting that these nanoparticles possess a high surface charge. This high surface charge indicates good colloidal stability, providing sufficient repulsive forces to prevent aggregation [61]. Such stability is crucial for maintaining the dispersion and functionality of nanoparticles in suspension.

The smooth spherical shape of the nanoparticles suggests that the synthesis process successfully produced uniform particles, which is often desired for consistent application performance. The absence of clumping or adhesion among the nanoparticles indicates a stable formulation. This stability is crucial for maintaining the discrete nature of the particles, which is important for their potential use in various applications. The high entrapment efficiency percentage indicates the effective encapsulation of both THQ and NTZ into the CsNPs. This influences drug release kinetics and overall formulation efficacy. Encapsulation in the CsNPs helps protect the loaded THQ and NTZ while providing biocompatible and biodegradable carrier properties. THQ/CsNPs and NTZ/CsNPs exhibited a biphasic release pattern characterized by an initial rapid burst followed by a slower, sustained release phase. This suggests that the drugs are initially released from the surface of the nanoparticles, followed by a more gradual diffusion from the core. The slower release rates of THQ and NTZ from the CsNPs compared to their free forms can be attributed to the entrapment of the drugs within the nanoparticle matrix. This entrapment likely hinders immediate release, allowing for a more controlled and sustained release over time. The initial burst release may be due to free THQ and NTZ localized on the nanoparticle surface, while the subsequent sustained release phase could arise from the diffusion of the drugs from the core as the chitosan matrix swells. This dual-phase release is advantageous for applications requiring immediate and prolonged drug availability.

The findings confirmed that THQ/CsNPs are therapeutically advantageous and economically viable compared to NTZ/CsNPs. The lower cost of THQ/CsNPs is achieved through careful optimization of the synthesis process, including reduced material usage and energy efficiency. This cost advantage, combined with the enhanced bioavailability and stability of THQ encapsulated in chitosan nanoparticles, positions THQ/CsNPs as a promising alternative to NTZ-based formulations for drug delivery applications.

The cytotoxicity results demonstrated a dose-dependent effect for all tested nanoparticles, with CsNPs showing the highest biocompatibility and minimal cytotoxicity, while THQ/CsNPs and NTZ/CsNPs exhibited increased cytotoxic effects. The encapsulation of bioactive compounds (thymoquinone and nitazoxanide) in CsNPs enhanced their cytotoxicity, likely due to improved cellular uptake and therapeutic efficacy. NTZ/CsNPs showed the highest cytotoxicity, suggesting their potential for applications requiring stronger antiproliferative effects. These findings highlight the versatility of CsNPs as a drug delivery platform, with their cytotoxic effects varying based on the loaded compounds. Further studies are recommended to optimize these formulations for therapeutic applications while ensuring safety and efficacy [58,62].

Regarding parasitological examination, our data showed that THQ/CsNPs had the highest oocyst shedding reduction compared to the corresponding infected control group and other treated groups on days 14 and 21 dpi (Table 1). Using chitosan nanoparticles alone showed a non-significant reduction compared to the infected control group. On the other hand, using thymoquinone alone, NTZ alone, or NTZ/CsNPs showed similar significant reductions. In this regard, our findings are consistent with previous research, including the results of Mohamed et al., who demonstrated that *Nigella sativa* conjugated with chitosan nanoparticles against cryptosporidiosis had a strong effect in reducing the amount of oocysts excreted in both immunocompetent and immunosuppressed individuals [63]. Additionally, these results are consistent with the findings of Moawad et al. [49], who reported the greatest percentages of reduction in oocyst shedding (41.39% and 67.33%) on days 11 and 19 dpi, respectively, upon treatment with NTZ-loaded CsNPs. Furthermore, it was revealed that using CsNPs alone had a non-significant effect on the mean oocyst count/g feces on days 11 and 19 dpi, with reductions of 4.08% and 7.68%, respectively [64].

The histological alterations in the intestine, liver, and spleen were evaluated to determine the effectiveness of various medication forms in treating intestinal and extra-intestinal cryptosporidiosis. In line with previous research, the histopathology results indicated that in immunosuppressed mice, the terminal segment of the ileum had the highest infection burden [65]. It has been proposed that favorable ileal conditions, such as biochemical parameters and the presence of specific receptors, aid in the parasite’s development. Additionally, Certad et al. discovered that the ileocecal area was the primary location for parasite localization and histological alterations [6].

In the present study, histopathological examination of sections from the intestinal, spleen, and liver tissues in the positive control group (non-treated) documented profound alterations in the villi architecture of the small intestine, consistent with *Cryptosporidium* infection. These alterations included loss of villous architecture, inflammatory cell infiltration, villous atrophy, and goblet cell hyperplasia. *Cryptosporidium* stages were observed along the brush border of the villi and in side crypts. Examination of liver sections revealed mononuclear inflammatory cell infiltration, acute cell swelling, hyperplasia of Kupffer cells, focal necrosis, and hepatocyte atrophy. Hematopoiesis occurred in the spleen, exhibiting lymphoid and vascular components [66]. Research on the effect of *C. parvum* infections on the spleen is limited. Mouse spleens challenged with *C. parvum* showed signs of inflammation but did not harbor the parasite developmental stages. Examination of the spleen showed severe depletion of lymphoid follicles and a marked presence of megakaryocytes. No significant differences were found between the infected group treated with blank chitosan nanoparticles and the infected group that was not treated. These findings are consistent with those of [67–70], who reported comparable histopathological alterations in infected animals, including inflammatory infiltration, villous atrophy, splenic depletion, and liver degradation. Similar histopathological findings were reported by Soufy et al. [71], who noted that *Cryptosporidium* parasites displaced brush borders, resulting in the shortening and broadening of the villi. Toxins released by the infection that causes direct damage to epithelial cells could account for villous atrophy [71].

A variable degree of improvement in histopathological changes in the small intestine, liver, and spleen tissues was detected following treatment with NTZ alone, NTZ-loaded chitosan nanoparticles, and thymoquinone alone. NTZ alone and thymoquinone alone showed partial improvement in histopathological changes in the small intestine, with persistent villous atrophy, inflammatory cell presence, and mucosal oocysts. Moreover, there was a partial improvement of lymphoid content in the spleen and mild improvement in liver architecture, with focal necrosis and multiple aggregations of inflammatory cells. This agrees with Sadek and El-Aswad [72], who reported moderate changes in mice treated with NTZ and severe pathological changes in the infected control group. Our data revealed that NTZ-loaded chitosan nanoparticles showed significantly more improvement in histopathological findings compared to NTZ alone or thymoquinone alone. These findings are consistent with those of Moawad et al. [64]. Improvement was evident by the return of a normal villous pattern and mild infiltration of inflammatory cells [64]. Numerous studies have documented similar histopathological changes in treated mice with NTZ-loaded chitosan nanoparticles [73–75].

In the current study, the group treated with thymoquinone loaded on chitosan nanoparticles showed the best results in histopathological aspects, manifested by regenerative attempts of enterocytes (re-epithelization), normal mucosa, goblet cells, and the return of the normal villous pattern with no inflammatory cells. Additionally, liver examination showed a normal histological pattern, and spleen examination revealed normal lymphoid follicles filled with numerous lymphocytes. The antiparasitic properties of thymoquinone loaded on chitosan nanoparticles were previously assessed against Leishmania [22] and Schistosomiasis [36]. Islamuddin et al. [22] confirmed that thymoquinone had significant leishmanicidal activity against *L. donovani*. The leishmanicidal effect was attributed to programmed cell death, evidenced by nuclear DNA nicking, phosphatidylserine exposure, and reduced mitochondrial membrane potential without adverse effects on the murine macrophage cell line [22].

Specific markers are used to detect true dysplastic changes, as atypical changes secondary to inflammation and infection could affect the epithelium of the gastrointestinal tract morphologically and simulate true dysplastic changes. In the present study, we used cyclin D1, a cell cycle regulator, to identify true dysplasia. Its evaluation as a nuclear marker was consistent, and we observed that it stained the nuclei in areas with dysplastic changes. However, normal areas were not stained by cyclin D1. In the current study, the control infected group and the blank chitosan nanoparticles group showed strong immunoreactivity, manifesting as brown granules inside the lining epithelium of villi and crypts. These findings agree with others [76,77], who suggested that abnormal upregulation of cyclin D1 may be an early event in intestinal carcinogenesis [76]. Different degrees of immunostaining were observed in other treated groups, with the best results detected in the group treated with thymoquinone loaded on chitosan nanoparticles, which showed negative immunoreactivity.

*Cryptosporidium parvum* infection and pathogenesis are controlled by the interaction of both Th1 (IFN-γ, TNF-α, and IL-12) and Th2 (IL-4 and IL-10) cytokine responses [78]. IFN-γ induces M1 macrophage expansion, induces ROS and NO production, and causes apoptosis [69]. According to our findings, infected non-treated mice secreted more pro-inflammatory cytokine (IFN-γ) than control mice, indicating the inflammatory reactions associated with *Cryptosporidium* infestation. This led to severe pathogenesis with no significant change in the level of Th2 (anti-inflammatory cytokines) compared to the healthy control group. Immune responses were modulated by THQ/CsNPs and NTZ/CsNPs therapies. In the early stage of infection, Group V and Group VII showed a significantly lower level of Th1 cytokines (IFN-γ) compared to the control-infected group. Later, more balanced responses through Th2 cytokines (IL-10) occurred, which increased significantly to limit intestinal pathology and facilitate cure. This result aligns with Abouel-Nour et al. [79] and Abdelgelil et al. [78]. THQ demonstrated an anti-inflammatory effect by suppressing the expression of pro-inflammatory and proliferative mediators like TNF-α, inducible nitric oxide, cyclooxygenases-2, 5-lipoxygenase, and cyclin D1 [80].

Thymoquinone exerts its effects mainly through the modulation of key immune signaling pathways. It has been shown to inhibit NF-κB and MAPK pathways, which are central to activating inflammatory cytokines (like TNF-α, IL-1, and IFN-γ). THQ has antioxidant properties and can modulate the JAK-STAT pathway, particularly influencing cytokine production (e.g., promoting IL-10 while suppressing pro-inflammatory cytokines like IFN-γ). Furthermore, THQ’s action is often more selective and targeted at specific immune responses, potentially enhancing anti-inflammatory pathways (like IL-10) while suppressing harmful pro-inflammatory responses [81].

The pharmacokinetic findings demonstrated that chitosan-based nanoparticle formulations significantly enhance drug absorption, retention, and elimination profiles compared to the free drug. THQ/CsNPs and NTZ/CsNPs consistently exhibited higher plasma concentrations across all time points, with earlier *T*_max_ values (2 hours) compared to free drugs (THQ, *T*_max_ = 4 hours; NTZ, *T*_max_ = 2 hours), indicating faster absorption facilitated by nanoparticle encapsulation. The sustained higher plasma concentrations observed for composite formulations at later time points, such as 12 and 24 hours, suggest extended therapeutic effects due to slower clearance or gradual drug release. At 12 hours, THQ/CsNPs maintained a concentration of 51.32 ± 5.9 µg/mL compared to free THQ at 4.88 ± 1.79 µg/mL, while NTZ/CsNPs retained 61.18 ± 6.49 µg/mL compared to free NTZ at 4.16 ± 2.71 µg/mL. Additionally, the prolonged half-life (*t*_1/2_) observed for NTZ/CsNPs (*t*_1/2 _= 10.09 ± 0.89 hours compared to *t*_1/2 _= 6.55 ± 0.54 hours for free NTZ) suggested that nanoparticle encapsulation slows metabolism or excretion processes, potentially reducing dosing frequency and extending therapeutic windows. However, compared to free drugs, the reduced AUC₀ → 24 values for composite formulations indicated faster systemic clearance or limited sustained release over time, likely due to an initial burst release followed by rapid elimination. These results highlight the potential of chitosan-based nanoparticles to improve bioavailability and modulate pharmacokinetic profiles for poorly soluble drugs like THQ and NTZ. However, further optimization is needed to enhance sustained release properties while minimizing rapid clearance observed during the study period [82].

## Conclusion

The present study documented that utilizing thymoquinone, a bioactive compound from *Nigella sativa*, has the potential to be considered an effective agent in cryptosporidiosis treatment. Loading thymoquinone onto chitosan nanoparticles improved its efficacy by reducing oocyst shedding, improving histological alterations, and regulating the immune response. The cytotoxicity results demonstrated that chitosan nanoparticles (CsNPs) exhibit excellent biocompatibility, while THQ/CsNPs and NTZ/CsNPs show enhanced cytotoxic effects. This points to the importance of therapeutics based on nanocomposites in cryptosporidiosis treatment. Nevertheless, more research is needed to clarify the mechanisms involved in these actions. CsNPs improved the pharmacokinetics of THQ and NTZ by increasing Cmax, accelerating absorption (Tmax = 2 hours for composite formulations), and extending half-life (t1/2= 10.09 ± 0.89 hours for NTZ/CsNPs), highlighting their promise as drug delivery systems.

### Future directions for enhancing thymoquinone bioavailability and clinical translation

Thymoquinone (THQ) holds significant promise as a therapeutic agent due to its diverse pharmacological properties; however, its clinical translation is hindered by challenges such as poor aqueous solubility, low bioavailability, hydrophobicity, and thermolabile nature. To overcome these limitations, future research should focus on innovative drug delivery systems and formulation strategies that enhance THQ’s bioavailability and stability without compromising its efficacy and safety. Nanotechnology-based delivery systems, such as liposomes, polymeric nanoparticles, and nanostructured lipid carriers (NLCs), offer potential solutions to improve solubility, stability, and targeted delivery. Similarly, lipid-based formulations like solid lipid nanoparticles and self-emulsifying drug delivery systems can enhance absorption and protect THQ from degradation. Prodrug development is another promising approach to optimize THQ’s pharmacokinetic profile by improving solubility and reducing rapid elimination. Additionally, exploring combination therapies with synergistic agents may amplify THQ’s therapeutic efficacy while minimizing required dosages. Finally, transitioning from preclinical studies to well-designed clinical trials is imperative to evaluate THQ’s efficacy and safety in human subjects, providing the necessary data for pharmaceutical development and regulatory approval. By addressing these challenges, future research can unlock THQ’s full therapeutic potential and facilitate its successful application in clinical settings.
